# 

**Published:** 1885-07

**Authors:** 


### CONTENTS:

Art. I—Southern Dental Association. Proceedings of the Annual Meeting held in New Orleans, La. in March, 1885. (Continued.)....................97
Art. II—On the Availibility of Certain Antiseptics in the Prophylactic Treatment of the Oral Cavity. By Prof. W. B. Miller, A. B., D. D. S. Berlin.......119
Art. III—The Odontological Society of Great Britain...........................126
Art IV—Dental Hygiene. By G. L. Curtis, D. D. S..................................134

### EDITORIAL, ETC.

The Reason Why the Dental Department of the University of Maryland Deems it Inexpedient to Join the National Association of Dental Faculties...136

### MONTHLY SUMMARY.

Death from Inhaling Nitrous Oxide..........................................138

Some Modifications of the Antiseptic Method...............................139

Crown Work.....................................................................141

Discard Oxyphosphates.......................................................142

New Capsicum Plaster.........................................................143

A Narrow Escape................................................................144

